# Accurate Reaction Probabilities for Translational Energies on Both Sides of the Barrier of Dissociative Chemisorption on Metal Surfaces

**DOI:** 10.1021/acs.jpclett.3c03408

**Published:** 2024-02-28

**Authors:** Nick Gerrits, Bret Jackson, Annemie Bogaerts

**Affiliations:** † Leiden Institute of Chemistry, Gorlaeus Laboratories, Leiden University, Post Office Box 9502, 2300 RA Leiden, Netherlands; ‡ Research Group PLASMANT, Department of Chemistry, University of Antwerp, Universiteitsplein 1, BE-2610, Wilrijk, Antwerp, Belgium; § Department of Chemistry, University of Massachusetts Amherst, Amherst, Massachusetts 01003, United States

## Abstract

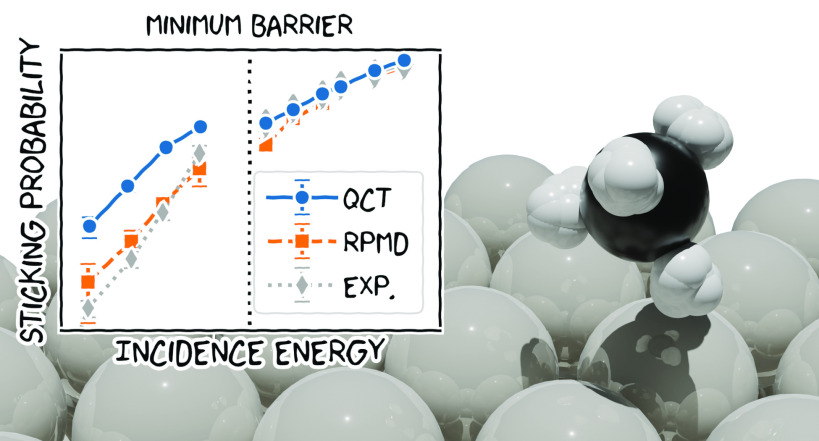

Molecular dynamics simulations are essential for a better understanding of dissociative chemisorption on metal surfaces, which is often the rate-controlling step in heterogeneous and plasma catalysis. The workhorse quasi-classical trajectory approach ubiquitous in molecular dynamics is able to accurately predict reactivity only for high translational and low vibrational energies. In contrast, catalytically relevant conditions generally involve low translational and elevated vibrational energies. Existing quantum dynamics approaches are intractable or approximate as a result of the large number of degrees of freedom present in molecule–metal surface reactions. Here, we extend a ring polymer molecular dynamics approach to fully include, for the first time, the degrees of freedom of a moving metal surface. With this approach, experimental sticking probabilities for the dissociative chemisorption of methane on Pt(111) are reproduced for a large range of translational and vibrational energies by including nuclear quantum effects and employing full-dimensional simulations.

Dissociative chemisorption (DC) on metal surfaces is often a rate-controlling state in heterogeneous catalysis.^1−3^ One of the major issues in simulating DC and improving our understanding is the employed dynamical model. Molecular dynamics simulations are often required because more approximate methods (e.g., static calculations and transition state theory) can only provide limited information and dynamical effects can cause significant deviation from predictions based on such calculations.^4^ For example, rovibrational excitation can affect the reactivity and reaction mechanism in a complex fashion as a result of features in the potential energy surface (PES) of the reaction.^5−9^ Also, as a result of surface atom motion, energy transfer between the molecule and the metal surface as well as temperature-dependent barrier height modulation can affect the reactivity considerably.^7,10,11^ Furthermore, the gold standard of obtaining barrier heights for DC and benchmarking theory is to perform molecular beam experiments and compare dynamical simulations to the experiments, because experiments cannot measure barrier heights directly.^4^ In short, dynamical simulations of DC on metal surfaces are of practical and fundamental interest.

Theory has become increasingly better at accurately predicting and reproducing experimental sticking probabilities for DC. Many challenges to the accuracy exist, e.g., the accuracy of the electronic structure theory,^4,12,13^ the limited tractability,^7,14−16^ and the breakdown of the Born–Oppenheimer approximation.^17−21^ Here, we focus on the employed dynamical model, where the quasi-classical trajectory (QCT) approach^22^ is ubiquitous. In this approach, the quantum mechanical rovibrational energy is imparted to the molecule, and the equations of motion are subsequently propagated in a classical fashion. Nowadays, the forces used to propagate the equations of motion are generally obtained from either a fit to or directly from the electronic structure theory. QCT has not only been successful in reproducing experimental gas phase reaction probabilities^23−25^ but also molecule–metal surface reaction probabilities.^4,12,16,26−28^ However, nuclear quantum effects (NQEs) are neglected in the QCT approach. This can affect the computed reaction probability when the translational energy of a molecule is near or lower than the barrier height in two major ways: The neglect of tunneling effects artificially lowers the reaction probability, whereas the artificial leakage of zero-point energy (ZPE) into the reaction coordinate increases the reaction probability. This is problematic for the prediction of reaction rates in heterogeneous catalysis, because the employed reaction conditions often involve translational energies lower than the barrier height. Another issue is that, for polyatomic molecules, the QCT approach has been seen to overestimate the reactivity when the vibrational temperature is high as a result of artificial intramolecular vibrational energy redistribution (IVR).^26^ Because the vibrational energy is not quantized in QCT (i.e., vibrational energy can flow continuously between different vibrational modes), IVR occurs too facile, especially when vibrational modes are excited. Also, the ZPE leakage occurs more readily when polyatomic instead of diatomic molecules are involved as a result of the increase in the number of vibrational modes. Therefore, the reactivity of polyatomic molecules under catalytically relevant conditions tends to be overestimated dramatically in QCT simulations. Moreover, in plasma catalysis, the translational energy tends to be lower combined with a considerably higher vibrational temperature compared to heterogeneous catalysis, making reliable dynamical simulations even more difficult.^29,30^ This is unfortunate because plasma catalysis has the potential to increase the efficiency of industrial processes and utilize green energy by combining heterogeneous catalysis with plasmas but lacks a fundamental understanding and, therefore, requires accurate simulations to develop mature plasma technology.^31−37^

Accurate wave packet quantum dynamics (QD) do include NQEs but generally scale badly with the number of degrees of freedom (DOFs) on top of the already considerably higher computational cost compared to QCT, severely limiting the number of DOFs and the quality of the QD basis set that can be treated, also in the foreseeable future.^38−40^ Recent developments try to include the full effect of surface atom motion, which dramatically increases the number of DOFs, in QD simulations in an affordable fashion.^41−44^ Unfortunately, such calculations are even more expensive than static surface calculations, and it is unclear how accurate they are in describing a fully moving surface interacting with a (polyatomic) molecule, where surface atom motion is often a non-negligible effect.

Ring polymer molecular dynamics (RPMD) poses an interesting alternative to wave packet QD.^45^ In RPMD, classical dynamics are extended approximately into a quantum regime through a path integral approach.^46^ By doing this, NQEs, such as tunneling and ZPE conservation, are included at a reasonable increase of cost (generally 1–2 orders of magnitude compared to QCT). RPMD has been successfully applied to, e.g., gas phase reactions,^47−49^ hydrogen diffusion on and NO desorption from metal surfaces,^50,51^ water,^52−55^ and H atom scattering from graphene.^56^ Furthermore, the RPMD approach has also been used to simulate the DC of H_2_ on Cu(111) and D_2_O on Ni(111), where it was observed that RPMD can accurately reproduce wave packet QD.^57^ Recently, DC rates of H_2_ on Pt(111) and Ag(111) have been (approximately) computed using RPMD rate theory,^58^ which yielded a qualitative improvement over classical rate theory. However, in both DC studies, the surface atoms were kept fixed in their ideal positions, neglecting any dynamical effects as a result of surface atom motion, such as barrier height modulation and energy transfer between the molecule and the metal surface. Furthermore, the latter study requires the molecule and metal surface to be in thermal equilibrium, whereas the reaction conditions in the molecular beam experiments that we simulate are not. In this work, we show, for the first time, that RPMD can accurately simulate DC on a moving metal surface for a large range of vibrational and translational energies, both below and above the minimum barrier height and where the system is not in thermal equilibrium.

Specifically, we choose the DC of methane on Pt(111) as a test case for our approach because the reaction dynamics are well-understood,^59,60^ a large amount of experimental data is available to benchmark theory,^27,61−66^ and it is a system where the ZPE and surface atom motion play an important and non-negligible role in the reactivity.^7,43,60,67,68^ It should be noted that CH_4_ contains considerably more ZPE and vibrational modes (≈1.2 eV and 9) than H_2_ (≈0.3 eV and 1) and D_2_O (≈0.4 eV and 3) and is therefore expected to suffer more from ZPE leakage and artificial IVR in QCT than the aforementioned systems previously investigated by Liu et al.^57^ Furthermore, it is one of the first molecule–metal surface reactions for which a chemically accurate density functional (DF) was found.^26,27,69^ QCT was able to reproduce the sticking probability of CHD_3_ on Pt(111) for both “laser-off” and “laser-on” conditions at incidence energies near or above the minimum barrier height with the use of a so-called specific reaction parameter^12^ (SRP) DF, i.e., the SRP32-vdW-DF1 DF,^26^ which is also used in this work. The laser-off conditions correspond to a vibrational Boltzmann distribution dependent upon the vibrational temperature. The laser-on conditions correspond to a vibrational Boltzmann distribution of which specific rovibrational states are excited, from which ultimately a rovibrational state-specific sticking probability can be extracted. In this work, we focus on the laser-off conditions, because vibrational state-specific RPMD is not trivial to perform (*vide infra*).

Because *ab initio* molecular dynamics (AIMD) are expensive and intractable for this system to perform sufficient RPMD calculations to obtain statistically significant results, we developed a high-dimensional neural network potential (HDNNP) for methane + Pt(111). The Behler–Parrinello^70^ approach is used because it has been shown to accurately describe DC of several molecules on metal surfaces.^7,8,15,16,71−73^ Moreover, we use the same approach as used in ref (7), in which an HDNNP was constructed for CHD_3_ + Cu(111) (see section S1 of the Supporting Information for more details). We have confirmed that the HDNNP accurately reproduces our density functional theory (DFT) calculations and previous AIMD calculations on CHD_3_ + Pt(111)^27^ (section S2 of the Supporting Information). For the RPMD, we take an approach similar to ref (57), but here, the surface atom motion is included as well. The vibrational initial conditions of the molecule are obtained by performing canonical *NVT* (constant number of particles, volume, and temperature) simulations in the gas phase with the so-called PIGLET approach,^74,75^ of which any translational and rotational motion is removed afterward because, from these simulations, we only require the vibrational positions and moments. For the initial conditions of the surface, separate *NVT* simulations are performed at the surface temperature to sample the positions and velocities of the surface atoms. Finally, we perform microcanonical *NVE* (constant number of particles, volume, and energy) simulations to simulate DC, by first adding translational and rotational motion to the center of mass of the molecule as well as reorienting the molecule according to its rotational state (see for example chapter 2 of ref (76)). The reader is referred to section S3 of the Supporting Information for additional details regarding the simulations. It should be noted that, opposite to what is usual in RPMD, the *NVE* simulations performed here are thermally not in equilibrium. Initially, the system contains several different “temperatures” or distributions. For the molecule, a different distribution for the vibration, rotation, and translation is used compared to thermal equilibrium, whereas the metal surface is in thermal equilibrium but notably different compared to the molecule. During the interaction between the molecule and the metal surface, the two exchange energy, after which, as a result of the conservation of energy instead of the temperature as well as the short time scale, the entire system should again be non-equilibrium. Fortunately, RPMD seems thus far to be well-suited for not just equilibrium but for non-equilibrium simulations as well.^49,56,57,77−79^ As we will show, RPMD seems to be well-suited to also treat specifically non-equilibrium reactive scattering of molecules from moving metal surfaces.

Figure 1 shows the sticking probability of methane on Pt(111). First, we look at incidence energies below the minimum barrier height (Figure 1A), where we compare to the experimental results of ref (66) using CH_4_. As expected, for incidence energies below the minimum barrier height, QCT overestimates the experimental sticking probability as a result of artificial ZPE leakage into the reaction coordinate. In contrast, RPMD yields accurate sticking probabilities in good agreement with the experiment.

**Figure 1:**
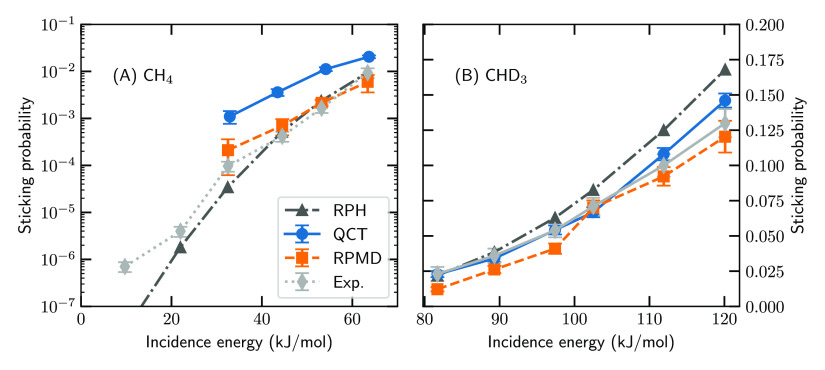
Sticking probability of (A) CH_4_ and (B) CHD_3_ on Pt(111). Shown are the RPH (black triangles), QCT (blue circles), RPMD (orange squares), and experimental^27,66^ (gray diamonds) results. Error bars indicate 68% confidence intervals.

Although the RPMD calculations are considerably cheaper than QD, in this work, they are still 2 orders of magnitude more expensive than QCT. Because calculations for lower incidence energies than performed here would also require considerably more trajectories (10^6^–10^8^ instead of 10^4^), it is intractable at present to compute RPMD sticking probabilities for lower incidence energies than those presented here. Future developments in machine-learned potentials and RPMD techniques combined with a general increase in computational resources should enable investigation of RPMD sticking probabilities at even lower incidence energies. Nevertheless, we can discuss how RPMD is expected to perform at incidence energies far below the minimum barrier height. For QCT, it is clear that the reactivity for translational energies below the minimum barrier height is always vastly overestimated as a result of artificial leakage of the ZPE into the reaction coordinate. Thus far, RPMD has been shown to preserve the ZPE during the reaction much better than QCT, making accurate predictions of the sticking probability at low incidence energies possible. At *E*_i_ = 33 kJ/mol, RPMD overestimates the experimental sticking slightly, but it should also be noted that the experimental result still falls well within the 1σ confidence interval of the RPMD result (the RPMD statistics here are severely limited, with only 4 trajectories out of 20 000 having reacted). Previous results also indicate that, in general, RPMD yields accurate reaction rates even in the deep tunneling regime.^80−84^ Moreover, the reactivity of methane at lower incidence energies is dominated by trajectories in which the molecule encounters a surface configuration that lowers the local barrier height considerably and not by tunneling or energy exchange between the molecule and metal surface.^7,11,60,67,68,85^ In fact, RPMD calculations employing a static ideal surface [i.e., the so-called Born–Oppenheimer static surface (BOSS) approximation, which approximates a *T*_s_ = 0 K surface but still includes the thermal lattice expansion corresponding to *T*_s_ = 500 K] yielded at *E*_i_ = 33 kJ/mol no reactive trajectories out a total of 20 000, in good agreement with previous experimental and theoretical results, showing a considerable increase in sticking at low incidence energy with the surface temperature.^61,63,65,85−87^ Exploratory calculations also suggest that the BOSS results match the moving surface results at higher incidence energies above the minimum barrier height, again in agreement with the experiment and theory. In short, both ZPE conservation and surface atom motion must be described correctly to provide accurate simulations for CH_4_ + Pt(111) at incidence energies below the minimum barrier height, which seems to be the case here. For these reasons, we expect RPMD to reproduce the experiments at even lower incidence energies as well. We hope that the aforementioned developments will allow for testing this hypothesis in the future.

For incidence energies above the minimum barrier height (Figure 1B), we compare to the experimental results of ref (27), where CHD_3_ is employed. Both QCT and RPMD yield good agreement with the experiment. Interestingly, at the highest incidence energies and concomitant vibrational temperatures, the agreement between QCT and the experiment is reduced, which has been previously attributed to artificial IVR.^26,27,88^ This effect is also more noticeable in this work compared to ref (27) as a result of the improved error margins that the usage of an HDNNP over AIMD can yield (see also Figure S4 of the Supporting Information). In contrast, the agreement between RPMD and the experiment does not deteriorate at the highest employed vibrational temperatures. This suggests that RPMD does not suffer or at least not as fast as QCT from artificial IVR.

We have also included reaction path Hamiltonian^85,89,90^ (RPH) results in Figure 1 as a QD benchmark for RPMD, given the success of the method in simulating reactivity of methane on several metal surfaces.^10,27,91,92^ Our RPH calculations for CHD_3_ on Pt(111) are described in the Supporting Information of ref (27) but were only published in ref (93) and not in the original paper. The CH_4_ results in Figure 1a are new and were computed for this work using the same PES data. The CH_4_ and CHD_3_ sticking probabilities are computed using the molecular beam parameters provided in Tables S3 and S4 of the Supporting Information, respectively. The RPH approach accurately reproduces the experiments at low incidence energies (Figure 1A). However, these computationally inexpensive QD simulations make several assumptions, in particular, that the reactive molecular trajectories lie close to the minimum energy path (MEP), justifying a harmonic approximation for the vibrational motion. Large translational energies can cause significant deviation from the MEP through the so-called bobsled effect, where the molecule needs to “turn” on a PES in late barrier systems but slides off the MEP as a result of too much translational energy.^94,95^ This, in turn, lowers the reactivity because the molecule needs to cross a higher barrier to dissociate.^7,60,96^ Because this effect is missing in the RPH approach, the reactivity is overestimated at large translational energies. Furthermore, high vibrational temperatures also cause the RPH approach to overestimate reactivity. As such, the applicability of the RPH approach is mostly limited to the quantum regime and might not be viewed as a more general workhorse approach. Nevertheless, the RPH approach was developed specifically for low translational and vibrational temperatures. Because the RPH results employ the same SRP-DF (i.e., SRP32-vdW-DF1) as the QCT and RPMD calculations performed here, it is promising that, in those conditions, the RPH and RPMD approaches yield similar sticking probabilities that are in good agreement with the experiment. This also suggests that the RPMD approach accurately includes NQEs, without the need for any system-specific *a priori* approximations.

Although RPMD seems to already be a considerable improvement over QCT, there are some remaining issues that need future attention. First, QCT has been extensively used to obtain vibrational state-specific data. However, the approach used here to generate the initial molecular vibrational conditions for RPMD yields a canonical ensemble that is dependent upon the vibrational temperature. This is excellent when we compare to supersonic molecular beam experiments under “laser-off” conditions or other catalytically relevant experiments, because the vibrational state distribution is the same as the distribution that we simulate. However, to simulate a specific vibrational state, an approach to generate accurate initial conditions for the RPMD simulations does not yet exist. Marjollet and co-workers used a harmonic approximation, a low vibrational temperature ensemble to mimic the vibrational ground state distribution, and an instantaneous kick along a particular vibrational mode to simulate a vibrationally excited molecule.^97−99^ However, tests for H_2_ indicate that this harmonic approximation yields a considerably different ZPE compared to the full-dimensional RPMD simulations, and it is to be expected that the energy of excited vibrations is described even worse.^100^ Therefore, to extend the applicability of the RPMD approach to the full capabilities of QCT, a new approach for generating vibrational state-specific initial conditions in RPMD is required.

Second, the current formulation of the RPMD Hamiltonian requires a single temperature. However, in our simulations of DC, the system is not in equilibrium; i.e., there is not a single temperature. Therefore, a choice for the temperature has to be made, but results are also somewhat dependent upon that choice.^100^ Marjollet and co-workers used various ways to partition the translational and vibrational energy of the molecule in such a way that an effective temperature is obtained, but how this might be done accurately remains unclear.^97−99^ Moreover, in this work, we also have surface DOFs, which complicates determining the effective temperature even further. We have performed a few exploratory calculations and saw some effect of the effective temperature, where decreasing the temperature increases the sticking probability predominantly at low incidence energies and effective temperatures considerably lower than the surface temperature. Because, at a lower effective temperature, the extended ring polymer system is more delocalized, tunneling effects increase as well, and thus, it can be expected that the sticking probability is primarily increased at incidence energies lower than the minimum barrier height combined with a low temperature in the Hamiltonian. Fortunately, in our case, the computed results do not seem to be very dependent upon the choice of the temperature as long as the effective temperature is higher than that of the surface (*T*_s_ = 500 K; see Figure S5 of the Supporting Information). This is in agreement with Li et al., who observed hardly a difference for the sticking of H_2_ on a static Pd(111) surface at *T* = 300 and 1052 K.^100^ Nevertheless, it is clear that future work should also focus on how to better approximate the temperature in non-equilibrium RPMD.

In short, we show that RPMD can accurately reproduce experimental sticking probabilities for the DC of methane on Pt(111) at incidence energies both below and above the minimum barrier height. Furthermore, we use for the first time a moving surface in RPMD, which can affect the reactivity considerably. The good agreement between RPMD and the experiment is achieved through remedying the artificial ZPE leakage of the molecule into the reaction coordinate when the translational energy of the molecule is near or below the minimum barrier height. The results also suggest that the accuracy of RPMD is not sensitive to the employed molecular vibrational temperature by also reducing artificial IVR. In contrast, the accuracy of the workhorse QCT approach is considerably more dependent upon both the translational and vibrational temperature. Considering the moderate increase in computational costs compared to other QD approaches, we believe that RPMD is a cost-effective approach to include NQEs in non-equilibrium simulations of the DC of molecules on metal surfaces. This inclusion of NQEs is especially important for catalytically relevant simulations with the reaction conditions often being low translational and high vibrational energies, for which the QCT approach is typically inaccurate.

## Supporting Information Available

Description of the construction and accuracy of the HDNNP (sections S1 and S2, respectively) and the molecular dynamics (section S3), distribution of energy errors of the HDNNP (Figure S1), elbow plot of the barrier of methane on Pt(111) (Figure S2), van der Waals well of methane on Pt(111) (Figure S3), QCT sticking probability obtained with DFT and the HDNNP (Figure S4), dependence of the RPMD sticking probability upon the effect of the temperature in the ring polymer Hamiltonian (Figure S5), parameters of the radial and angular symmetry functions in the HDNNP (Tables S1 and S2, respectively), and molecular beam parameters for CH_4_ and CHD_3_ (Tables S3 and S4, respectively) (PDF)
Transparent Peer Review report available (PDF)
