# Supplementary material for: Accurate Reaction Probabilities for Translational Energies on Both Sides of the Barrier of Dissociative Chemisorption on Metal Surfaces
Source: J Phys Chem Lett. 2024 Feb 28;15(9):2566–72. doi: 10.1021/acs.jpclett.3c03408 (PMC10926167; doi:10.1021/acs.jpclett.3c03408)
Supplement: Supplementary file 1 — jz3c03408_si_001.pdf [file jz3c03408_si_001.pdf]

Supporting Information for: Accurate Reaction Probabilities for Translational Energies on Both Sides of the Barrier of Dissociative Chemisorption on Metal Surfaces

Nick Gerrits*[1,2], Bret Jackson[3], and Annemie Bogaerts[2]

[1]Leiden Institute of Chemistry, Leiden University, Gorlaeus Laboratories, P.O. Box 9502, 2300 RA Leiden, The Netherlands
[2]Research Group PLASMANT, Department of Chemistry, University of Antwerp, Universiteitsplein 1, BE-2610, Wilrijk, Antwerp, Belgium
[3]Department of Chemistry, University of Massachusetts Amherst, Amherst, MA 01003, USA

*email: n.gerrits@lic.leidenuniv.nl

Contents

S1 Construction of the Neural Network Potential **S5**

S2 Accuracy of the Neural Network Potential **S6**

S3 Molecular Dynamics **S6**

List of Figures

S1 Distribution of the absolute energy errors of the HDNNP compared to the DFT total energy. The data is taken from the training and test data sets. The dashed line indicates chemical accuracy, i.e., 4.2 kJ/mol. S11

S2 Elbow plot of the transition state of methane on Pt(111) as a function of Z_C and r, while keeping all other DOFs fixed at their minimum TS values. Contour lines are drawn at an interval of 10 kJ/mol between 60 and 100 kJ/mol, where red and blue indicate data obtained with the HDNNP and DFT, respectively. The dashed lines indicate the MEP in the shown reduced dimensionality and the black square indicates the highest point along the MEP. S11

S3 Van der Waals well of methane on Pt(111) with 1 (blue), 2 (orange), or 3 (grey) H atoms pointing towards the surface as a function of the distance Z_C between the carbon atom and the surface, while keeping methane fixed to its ideal gas phase geometry. The solid and dashed lines indicate results from the HDNNP and DFT, respectively. The gas phase is taken as the reference energy. S12

S4 Sticking probability of CHD_3 on Pt(111) as a function of incidence energy. Blue and orange circles indicate QCT results from DFT[1] and the HDNNP, respectively, and the grey diamonds indicate experimental results. The error bars represent confidence intervals of 68%. S12

S5 Sticking probability of CH_4 on Pt(111) as a function of incidence energy. Open blue and closed orange circles indicate RPMD results employing an effective temperature of 500 K and 1000 K, respectively, in the ring Hamiltonian. The closed grey diamonds indicate experimental results. The error bars represent confidence intervals of 68%. S13

List of Tables

S1 Parameters used for the radial symmetry functions (see Eq. S2) describing the interaction of the reference atom (Ref.) with its neighbouring atoms (Neighb.) within the cut-off radius. S14

S2 Parameters used for the angular symmetry functions (see Eq. S3) describing the interaction of the reference atom (Ref.) with its neighbouring atoms (Neighb. 1 and 2) within the cut-off radius. S15

S3 Molecular beam parameters from Ref.[26] (obtained via private communication) that describe the simulated CH_4 velocity distributions. The incidence energy E_i and the FWHM parameter were determined through time-of-flight measurements, whereas the stream velocity v_0 and width parameter α were determined through a fit to the aforementioned parameters using Eq. S4. . . . . S16

S4 Molecular beam parameters from Ref.[1] that describe the simulated CHD_3 velocity distributions. The stream velocity v_0 and width parameter α were determined through time-of-flight measurements S16

S1 Construction of the Neural Network Potential

The DFT calculations are performed with a computational setup that is identical to the one used in Ref.[1]. The initial data set for the fitting of the high-dimensional neural network potential (HDNNP) is obtained in the same manner as described in Ref.[2], extracting 28 335 structures from the AIMD data of Ref[1]. The vibrational and surface motion is additionally sampled by generating 4000 structures with an elevated vibrational ($T_{\mathrm{vib}} = 1200\,\mathrm{K}$) and surface ($T_{\mathrm{s}} = 1000\,\mathrm{K}$) temperature, but with the lattice expansion coefficient kept fixed at $T_{\mathrm{s}} = 500\,\mathrm{K}$. Finally, extrapolation errors are detected by running (RP)MD with the incomplete HDNNP, after which additional DFT calculations are performed and added to the training data set of the HDNNP. This yields a total amount of 48 517 structures used in the training of the HDNNP. These structures are finally randomly split into a training and testing data set, using a ratio of 90/10.

The training of the HDNNP and the static PES calculations are performed with RuNNer[3–5], where the latter is performed indirectly by using ASE[6] as an interface. Quasi-classical trajectory (QCT) simulations[7] are performed with LAMMPS[8,9], whereas RPMD simulations are performed with i-Pi[10,11], which is still interfaced with LAMMPS to obtain the forces.

The local environment of an atom is defined by the following cut-off function

$$f_c(R) = \begin{cases} \frac{1}{2}\left[\cos\left(\pi \frac{R}{R_c}\right) + 1\right] & R < R_c \\ 0 & R \geq R_c, \end{cases} \tag{S1}$$

where only atomic contributions within the cut-off radius are taken into account, here taken to be $13\,\mathrm{a}_0$. The radial symmetry functions (effectively two-body interactions) are[12,13]

$$G_i^{\mathrm{rad}} = \sum_{j \neq i} e^{-\eta (R_{i,j} - R_s)^2} f_c(R_{i,j}), \tag{S2}$$

where $R_{i,j}$ is the internuclear distance between atoms i and j, and η and R_s are parameters characterizing the function form, where $R_s = 0$ in this work. Furthermore, the angular symmetry functions (effectively three-body interactions) are taken as[12,13]

$$G_i^{\mathrm{ang}} = 2^{1-\zeta} \sum_{j,k \neq i} (1 + \lambda \cos\theta_{i,j,k})^{\zeta} e^{-\eta (R_{i,j} + R_{i,k} + R_{j,k})} f_c(R_{i,j}) f_c(R_{i,k}) f_c(R_{j,k}), \tag{S3}$$

where $\theta_{i,j,k} = \frac{R_{i,j} \cdot R_{i,k}}{R_{i,j} R_{i,k}}$, and η, ζ and λ are parameters characterizing the function form, where $\eta = 0$ in this work. The parameters used for the radial and angular symmetry functions are given in Tables S1 and S2.

S2 Accuracy of the Neural Network Potential

The RMSE of the test training data set is 0.6 meV/atom (total of 28.7 meV) and 24 meV/Å for the energies and forces, respectively. Furthermore, 85% of the test and training data set energies are reproduced within chemical accuracy (i.e., 4.2 kJ/mol), and 94% within two times chemical accuracy (Figure S1). In general, the structures that yield the largest errors tend to be very high-energy data that are likely to have only a (very) limited effect on the RPMD. The DFT minimum barrier height at $T_s = 500$ K (but keeping the surface atoms fixed at their ideal positions) is 78.4 kJ/mol, which is well reproduced by the HDNNP with 79.3 kJ/mol. In Figure S2, the potential energy surface surrounding the minimum barrier geometry (i.e., only the height of the carbon atom (Z_C) and the dissociating bond length r are varied, while all other degrees of freedom (DOFs) are kept fixed at their minimum barrier geometry values) as obtained with the HDNNP and DFT are in excellent agreement. Likewise, the agreement between the HDNNP and DFT for the van der Waals well in Figure S3 is excellent. Finally, the HDNNP and DFT are in good agreement for the sticking probabilities of CHD_3 as computed with the QCT approach (Figure S4).

S3 Molecular Dynamics

The QCT simulations are performed in the same way as in Ref.[1], with the notable exception that in this work the forces are obtained from the HDNNP instead of directly from DFT and the propagation of the equations of motion is performed in LAMMPS instead of VASP[14–20]. As such, we will only discuss the details relevant to the RPMD simulations.

The NVT simulations for the surface and molecular vibrational initial conditions employ the PIGLET approach[21,22], where the temperature in the RPMD Hamiltonian is always equal to that of the thermostat. Compared to QCT, the time step is reduced from 0.4 to 0.1 fs. In the NVE simulations, the system is no longer in equilibrium and therefore we need to make a choice for the effective temperature in the ring polymer Hamiltonian, here taken to be 1000 K. Figure S5 shows that, even for incidence energies well below the minimum barrier height, employing a lower effective temperature (500 K) hardly affects the sticking probability. Furthermore, we use 24 beads, which yields converged vibrational energies in the molecule. It should also be noted that in QCT the vibrational states are sampled by performing a 1D NVE simulation along each mode, from which the initial displacement (compared to the equilibrium geometry) and concomitant velocity is selected by randomly sampling the phase of the vibration. In contrast, in RPMD we perform a 15D NVT simulation (the 6 translational and rotational DOFs are removed afterwards) to obtain the vibrational positions and velocities. The vibrational temperature is

equal to the nozzle temperature. These simulations are first equilibrated for 5 ps and then propagated for another 5 ps, from which snapshots are taken every 10 fs. Also, for QCT the surface atom motion is sampled using a harmonic oscillator model and NVE simulations,[23–25] whereas for RPMD we simply use NVT simulations. For the surface motion, it is sufficient to use a time step of 1.0 fs, a total simulation time of 2 ps, and take a snapshot at each time step during the second half of the simulation. In all simulations we take the surface temperature T_s as 500 K. Although this is correct for the comparison with the CHD_3 experiments[1], the CH_4 experiments[26] have been performed with $T_\mathrm{s} = 600$ K. Fortunately, the difference in temperature is small and should have only a limited effect on the computed sticking probability.[27–31] To reduce the computational costs, the RPMD simulations use a slightly reduced reaction threshold (2.2 Å instead of 3 Å) compared to QCT, but this does not affect the results. For each sticking probability, between 2000 and 20 000 trajectories have been run. Moreover, the simulated rotational state of CH_4 and CHD_3 is $J = 0$ (i.e., the rotational ground state), which is appropriate for the simulation of supersonic molecular beams of methane[1,32]. The center of mass velocity v of the molecule in the molecular beam is given by the flux weighted probability distribution[33,34]

$$f\left(v; T_\mathrm{n}\right)\,\mathrm{d}v = A v^3 e^{-(v-v_0)^2/\alpha^2}\,\mathrm{d}v, \tag{S4}$$

where T_n is the nozzle temperature, A is a normalization constant, v_0 is the stream velocity, and α is the width of the distribution. The parameters for CH_4[26] and CHD_3[1] are provided in Tables S3 and S4, where for CH_4 Eq. S4 is fit to E_i and the FWHM to obtain v_0 and α. Finally, the reaction outcome is determined by computing positions and bond lengths of the centroid.

References

(1) Migliorini, D.; Chadwick, H.; Nattino, F.; Gutiérrez-González, A.; Dombrowski, E.; High, E. A.; Guo, H.; Utz, A. L.; Jackson, B.; Beck, R. D.; Kroes, G.-J. Surface Reaction Barriometry: Methane Dissociation on Flat and Stepped Transition-Metal Surfaces. *J. Phys. Chem. Lett.* **2017**, *8*, 4177–4182.

(2) Gerrits, N.; Shakouri, K.; Behler, J.; Kroes, G.-J. Accurate Probabilities for Highly Activated Reaction of Polyatomic Molecules on Surfaces Using a High-Dimensional Neural Network Potential: CHD_3 + Cu(111). *J. Phys. Chem. Lett.* **2019**, *10*, 1763–1768.

(3) Behler, J. Constructing High-Dimensional Neural Network Potentials: A Tutorial Review. *Int. J. Quantum Chem.* **2015**, *115*, 1032–1050.

(4) Behler, J. First Principles Neural Network Potentials for Reactive Simulations of Large Molecular and Condensed Systems. *Angew. Chem. Int. Ed.* **2017**, *56*, 12828–12840.

(5) Behler, J. RuNNer - A Neural Network Code for High-Dimensional Neural Network Potential-Energy Surfaces; Universität Göttingen, http://www.uni-goettingen.de/de/560580.html, 2018.

(6) Larsen, A. H. et al. The Atomic Simulation Environment—a Python Library for Working with Atoms. *J. Phys.: Condens. Matter* **2017**, *29*, 273002.

(7) Karplus, M.; Porter, R. N.; Sharma, R. D. Exchange Reactions with Activation Energy. I. Simple Barrier Potential for (H, H_2). *J. Chem. Phys.* **1965**, *43*, 3259–3287.

(8) Plimpton, S. Fast Parallel Algorithms for Short-Range Molecular Dynamics. *J. Comput. Phys.* **1995**, *117*, 1–19.

(9) Singraber, A.; Behler, J.; Dellago, C. Library-Based LAMMPS Implementation of High-Dimensional Neural Network Potentials. *J. Chem. Theory Comput.* **2019**, *15*, 1827–1840.

(10) Ceriotti, M.; More, J.; Manolopoulos, D. E. I-PI: A Python Interface for Ab Initio Path Integral Molecular Dynamics Simulations. *Comput. Phys. Commun.* **2014**, *185*, 1019–1026.

(11) Kapil, V. et al. I-PI 2.0: A Universal Force Engine for Advanced Molecular Simulations. *Comput. Phys. Commun.* **2019**, *236*, 214–223.

(12) Behler, J.; Parrinello, M. Generalized Neural-Network Representation of High-Dimensional Potential-Energy Surfaces. *Phys. Rev. Lett.* **2007**, *98*, 146401.

(13) Behler, J. Atom-Centered Symmetry Functions for Constructing High-Dimensional Neural Network Potentials. *J. Chem. Phys.* **2011**, *134*, 074106.

(14) Kresse, G.; Hafner, J. Ab Initio Molecular-Dynamics Simulation of the Liquid-Metal–Amorphous-Semiconductor Transition in Germanium. *Phys. Rev. B* **1994**, *49*, 14251–14269.

(15) Kresse, G.; Hafner, J. Ab Initio Molecular Dynamics for Liquid Metals. *Phys. Rev. B* **1993**, *47*, 558–561.

(16) Kresse, G.; Furthmüller, J. Efficient Iterative Schemes for Ab Initio Total-Energy Calculations Using a Plane-Wave Basis Set. *Phys. Rev. B* **1996**, *54*, 11169–11186.

(17) Kresse, G.; Furthmüller, J. Efficiency of Ab-Initio Total Energy Calculations for Metals and Semiconductors Using a Plane-Wave Basis Set. *Comput. Mater. Sci.* **1996**, *6*, 15–50.

(18) Kresse, G.; Joubert, D. From Ultrasoft Pseudopotentials to the Projector Augmented-Wave Method. *Phys. Rev. B* **1999**, *59*, 1758–1775.

(19) Román-Pérez, G.; Soler, J. M. Efficient Implementation of a van Der Waals Density Functional: Application to Double-Wall Carbon Nanotubes. *Phys. Rev. Lett.* **2009**, *103*, 096102.

(20) Klimeš, J.; Bowler, D. R.; Michaelides, A. Van Der Waals Density Functionals Applied to Solids. *Phys. Rev. B* **2011**, *83*, 195131.

(21) Ceriotti, M.; Bussi, G.; Parrinello, M. Nuclear Quantum Effects in Solids Using a Colored-Noise Thermostat. *Phys. Rev. Lett.* **2009**, *103*, 030603.

(22) Ceriotti, M.; Manolopoulos, D. E. Efficient First-Principles Calculation of the Quantum Kinetic Energy and Momentum Distribution of Nuclei. *Phys. Rev. Lett.* **2012**, *109*, 100604.

(23) Nattino, F.; Díaz, C.; Jackson, B.; Kroes, G.-J. Effect of Surface Motion on the Rotational Quadrupole Alignment Parameter of D_2 Reacting on Cu(111). *Phys. Rev. Lett.* **2012**, *108*, 236104.

(24) Nattino, F.; Migliorini, D.; Kroes, G.-J.; Dombrowski, E.; High, E. A.; Killelea, D. R.; Utz, A. L. Chemically Accurate Simulation of a Polyatomic Molecule-Metal Surface Reaction. *J. Phys. Chem. Lett.* **2016**, *7*, 2402–2406.

(25) Gerrits, N.; Migliorini, D.; Kroes, G.-J. Dissociation of CHD_3 on Cu(111), Cu(211), and Single Atom Alloys of Cu(111). *J. Chem. Phys.* **2018**, *149*, 224701.

(26) Bisson, R.; Sacchi, M.; Dang, T. T.; Yoder, B.; Maroni, P.; Beck, R. D. State-Resolved Reactivity of $CH_4(2\nu_3)$ on Pt(111) and Ni(111): Effects of Barrier Height and Transition State Location. *J. Phys. Chem. A* **2007**, *111*, 12679–12683.

(27) Schoofs, G. R.; Arumainayagam, C. R.; McMaster, M. C.; Madix, R. J. Dissociative Chemisorption of Methane on Pt(111). *Surf. Sci.* **1989**, *215*, 1–28.

(28) Oakes, D. J.; McCoustra, M. R.; Chesters, M. A. Dissociative Adsorption of Methane on Pt(111) Induced by Hyperthermal Collisions. *Faraday Discuss.* **1993**, *96*, 325–336.

(29) Valden, M.; Xiang, N.; Pere, J.; Pessa, M. Dissociative Chemisorption of Methane on Clean and Oxygen Precovered Pt(111). *Appl. Surf. Sci.* **1996**, *99*, 83–89.

(30) Tiwari, A. K.; Nave, S.; Jackson, B. The Temperature Dependence of Methane Dissociation on Ni(111) and Pt(111): Mixed Quantum-Classical Studies of the Lattice Response. *J. Chem. Phys.* **2010**, *132*, 134702.

(31) Guo, H. The Dissociative Chemisorption of Methane and Its Isotopologues on Metal Surfaces, Ph.D. Thesis, University of Massachusetts Amherst, 2018.

(32) Juurlink, L. B.; Smith, R. R.; Utz, A. L. The Role of Rotational Excitation in the Activated Dissociative Chemisorption of Vibrationally Excited Methane on Ni(100). *Faraday Discuss.* **2000**, *117*, 147–160.

(33) Auerbach, D. J. In *Atomic and Molecular Beam Methods*, Scoles, G., Ed.; Oxford University Press: 1988; Vol. 1, pp 362–379.

(34) Michelsen, H. A.; Auerbach, D. J. A Critical Examination of Data on the Dissociative Adsorption and Associative Desorption of Hydrogen at Copper Surfaces. *J. Chem. Phys.* **1991**, *94*, 7502–7520.

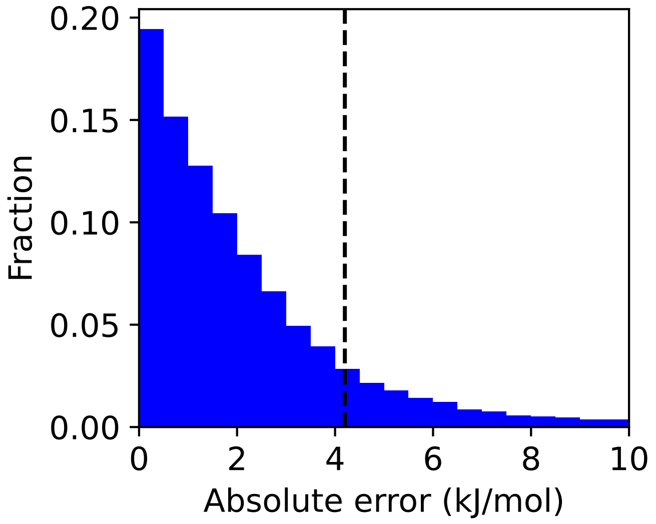

Figure S1: Distribution of the absolute energy errors of the HDNNP compared to the DFT total energy. The data is taken from the training and test data sets. The dashed line indicates chemical accuracy, i.e., 4.2 kJ/mol.

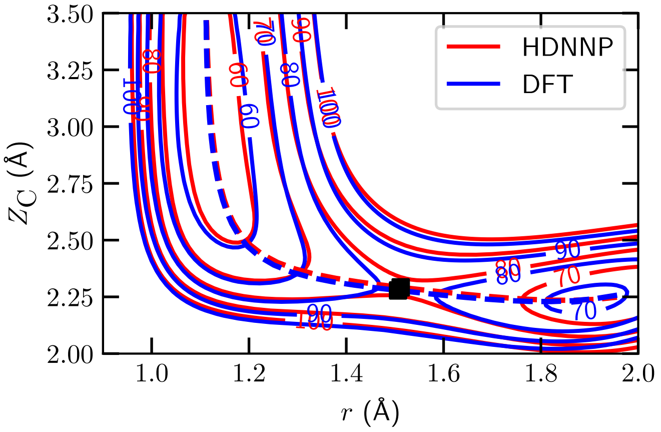

Figure S2: Elbow plot of the transition state of methane on Pt(111) as a function of Z_C and r, while keeping all other DOFs fixed at their minimum TS values. Contour lines are drawn at an interval of 10 kJ/mol between 60 and 100 kJ/mol, where red and blue indicate data obtained with the HDNNP and DFT, respectively. The dashed lines indicate the MEP in the shown reduced dimensionality and the black square indicates the highest point along the MEP.

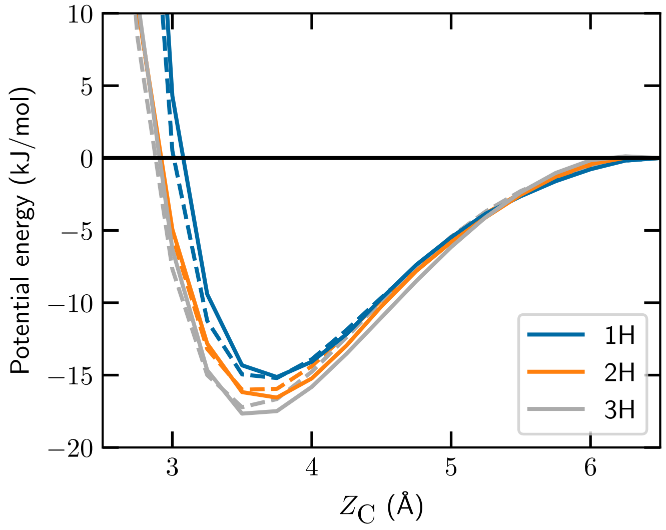

Figure S3: Van der Waals well of methane on Pt(111) with 1 (blue), 2 (orange), or 3 (grey) H atoms pointing towards the surface as a function of the distance Z_C between the carbon atom and the surface, while keeping methane fixed to its ideal gas phase geometry. The solid and dashed lines indicate results from the HDNNP and DFT, respectively. The gas phase is taken as the reference energy.

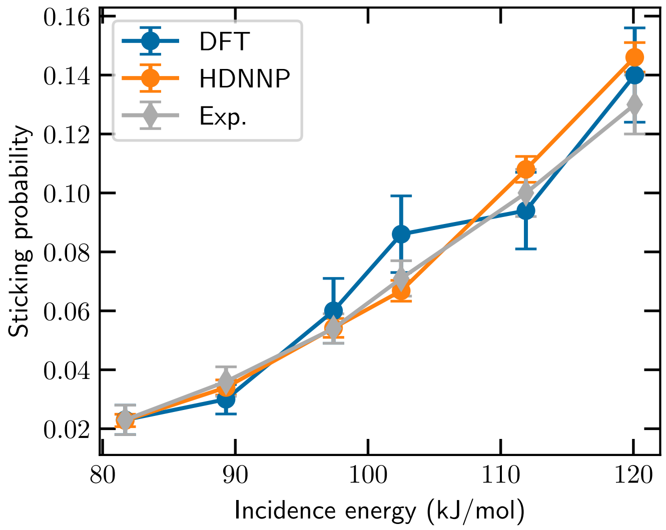

Figure S4: Sticking probability of CHD_3 on Pt(111) as a function of incidence energy. Blue and orange circles indicate QCT results from DFT[1] and the HDNNP, respectively, and the grey diamonds indicate experimental results. The error bars represent confidence intervals of 68%.

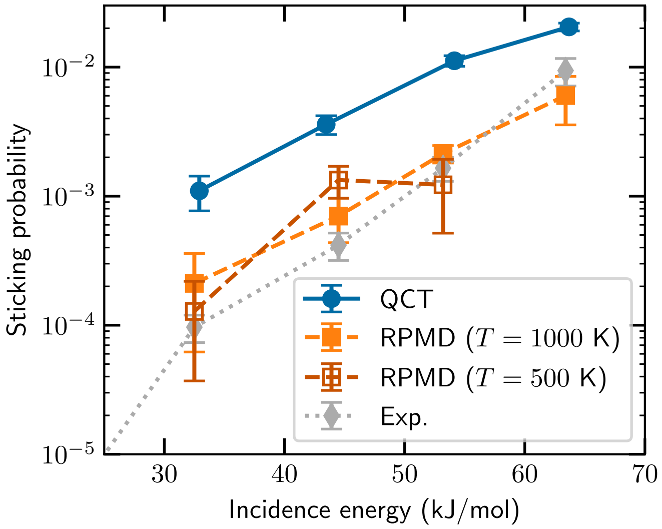

Figure S5: Sticking probability of CH_4 on Pt(111) as a function of incidence energy. Open blue and closed orange circles indicate RPMD results employing an effective temperature of 500 K and 1000 K, respectively, in the ring Hamiltonian. The closed grey diamonds indicate experimental results. The error bars represent confidence intervals of 68%.

Table S1: Parameters used for the radial symmetry functions (see Eq. S2) describing the interaction of the reference atom (Ref.) with its neighbouring atoms (Neighb.) within the cut-off radius.

Ref.	Neighb.	η	Ref.	Neighb.	η	Ref.	Neighb.	η
H	C	0.000	Pt	C	0.000	C	H	0.000
H	C	0.007	Pt	C	0.005	C	H	0.007
H	C	0.018	Pt	C	0.011	C	H	0.018
H	C	0.036	Pt	C	0.021	C	H	0.036
H	C	0.068	Pt	C	0.034	C	H	0.068
H	C	0.130	Pt	C	0.055	C	H	0.130
H	C	0.270	Pt	C	0.088	C	H	0.270
H	C	0.700	Pt	C	0.145	C	H	0.700
			Pt	C	0.260			
			Pt	C	0.500			
H	H	0.000	Pt	H	0.000	C	Pt	0.000
H	H	0.007	Pt	H	0.005	C	Pt	0.005
H	H	0.018	Pt	H	0.012	C	Pt	0.011
H	H	0.035	Pt	H	0.022	C	Pt	0.021
H	H	0.065	Pt	H	0.037	C	Pt	0.034
H	H	0.120	Pt	H	0.060	C	Pt	0.055
H	H	0.240	Pt	H	0.098	C	Pt	0.088
H	H	0.550	Pt	H	0.165	C	Pt	0.145
			Pt	H	0.290	C	Pt	0.260
			Pt	H	0.600	C	Pt	0.500
H	Pt	0.000	Pt	Pt	0.000			
H	Pt	0.005	Pt	Pt	0.005			
H	Pt	0.012	Pt	Pt	0.012			
H	Pt	0.022	Pt	Pt	0.022			
H	Pt	0.037	Pt	Pt	0.037			
H	Pt	0.060	Pt	Pt	0.060			
H	Pt	0.098	Pt	Pt	0.098			
H	Pt	0.165	Pt	Pt	0.165			
H	Pt	0.290	Pt	Pt	0.300			
H	Pt	0.600						

Table S2: Parameters used for the angular symmetry functions (see Eq. S3) describing the interaction of the reference atom (Ref.) with its neighbouring atoms (Neighb. 1 and 2) within the cut-off radius.

Ref.	Neighb. 1	Neighb. 2	λ	ζ	Ref.	Neighb. 1	Neighb. 2	λ	ζ	Ref.	Neighb. 1	Neighb. 2	λ	ζ
H	H	H	1	5.00	Pt	H	H	1	1.05	C	H	H	1	1.20
H	H	H	1	7.50	Pt	H	H	1	1.70	C	H	H	1	1.80
H	H	H	1	12.00	Pt	H	H	1	3.00	C	H	H	1	3.00
H	H	H	1	20.00	Pt	H	H	1	6.00	C	H	H	1	5.30
H	H	H	1	40.00	Pt	H	H	1	15.00	C	H	H	1	10.00
					Pt	H	H	1	68.00					
H	H	H	-1	1.30	Pt	H	H	-1	1.00	C	H	H	-1	5.00
H	H	H	-1	1.90	Pt	H	H	-1	1.27	C	H	H	-1	8.00
H	H	H	-1	2.80	Pt	H	H	-1	1.65	C	H	H	-1	13.00
H	H	H	-1	4.30	Pt	H	H	-1	2.15	C	H	H	-1	23.00
H	H	H	-1	7.00	Pt	H	H	-1	2.90	C	H	H	-1	50.00
					Pt	H	H	-1	4.00					
H	C	H	1	9.00	Pt	H	C	1	1.05	C	H	Pt	1	1.05
H	C	H	1	13.00	Pt	H	C	1	1.70	C	H	Pt	1	1.70
H	C	H	1	21.00	Pt	H	C	1	3.00	C	H	Pt	1	3.00
H	C	H	1	38.00	Pt	H	C	1	6.00	C	H	Pt	1	6.00
H	C	H	1	80.00	Pt	H	C	1	15.00	C	H	Pt	1	15.00
					Pt	H	C	1	68.00	C	H	Pt	1	68.00
H	C	H	-1	1.00	Pt	H	C	-1	1.00	C	H	Pt	-1	1.00
H	C	H	-1	1.30	Pt	H	C	-1	1.27	C	H	Pt	-1	1.27
H	C	H	-1	1.80	Pt	H	C	-1	1.65	C	H	Pt	-1	1.65
H	C	H	-1	2.50	Pt	H	C	-1	2.15	C	H	Pt	-1	2.15
H	C	H	-1	3.50	Pt	H	C	-1	2.90	C	H	Pt	-1	2.90
					Pt	H	C	-1	4.00	C	H	Pt	-1	4.00
H	Pt	H	1	1.05	Pt	Pt	H	1	1.05	C	Pt	Pt	1	1.05
H	Pt	H	1	1.70	Pt	Pt	H	1	1.70	C	Pt	Pt	1	1.70
H	Pt	H	1	3.00	Pt	Pt	H	1	3.00	C	Pt	Pt	1	3.00
H	Pt	H	1	6.00	Pt	Pt	H	1	6.00	C	Pt	Pt	1	6.00
H	Pt	H	1	15.00	Pt	Pt	H	1	15.00	C	Pt	Pt	1	15.00
H	Pt	H	1	68.00	Pt	Pt	H	1	68.00	C	Pt	Pt	1	68.00
H	Pt	H	-1	1.05	Pt	Pt	H	-1	1.05	C	Pt	Pt	-1	1.00
H	Pt	H	-1	1.70	Pt	Pt	H	-1	1.70	C	Pt	Pt	-1	1.27
H	Pt	H	-1	3.00	Pt	Pt	H	-1	3.00	C	Pt	Pt	-1	1.65
H	Pt	H	-1	6.00	Pt	Pt	H	-1	6.00	C	Pt	Pt	-1	2.15
H	Pt	H	-1	15.00	Pt	Pt	H	-1	15.00	C	Pt	Pt	-1	2.90
H	Pt	H	-1	68.00	Pt	Pt	H	-1	68.00	C	Pt	Pt	-1	4.00
H	Pt	C	1	1.05	Pt	Pt	C	1	1.05					
H	Pt	C	1	1.70	Pt	Pt	C	1	1.70					
H	Pt	C	1	3.00	Pt	Pt	C	1	3.00					
H	Pt	C	1	6.00	Pt	Pt	C	1	6.00					
H	Pt	C	1	15.00	Pt	Pt	C	1	15.00					
H	Pt	C	1	68.00	Pt	Pt	C	1	68.00					
H	Pt	C	-1	1.00	Pt	Pt	C	-1	1.05					
H	Pt	C	-1	1.27	Pt	Pt	C	-1	1.70					
H	Pt	C	-1	1.65	Pt	Pt	C	-1	3.00					
H	Pt	C	-1	2.15	Pt	Pt	C	-1	6.00					
H	Pt	C	-1	2.90	Pt	Pt	C	-1	15.00					
H	Pt	C	-1	4.00										
H	Pt	Pt	1	1.05	Pt	Pt	Pt	1	1.05					
H	Pt	Pt	1	1.70	Pt	Pt	Pt	1	1.70					
H	Pt	Pt	1	3.00	Pt	Pt	Pt	1	3.00					
H	Pt	Pt	1	6.00	Pt	Pt	Pt	1	6.00					
H	Pt	Pt	1	15.00	Pt	Pt	Pt	1	15.00					
H	Pt	Pt	1	68.00	Pt	Pt	Pt	1	68.00					
H	Pt	Pt	-1	1.00	Pt	Pt	Pt	-1	1.05					
H	Pt	Pt	-1	1.27	Pt	Pt	Pt	-1	1.70					
H	Pt	Pt	-1	1.65	Pt	Pt	Pt	-1	3.00					
H	Pt	Pt	-1	2.15	Pt	Pt	Pt	-1	6.00					
H	Pt	Pt	-1	2.90	Pt	Pt	Pt	-1	15.00					
H	Pt	Pt	-1	4.00										

Table S3: Molecular beam parameters from Ref.[26] (obtained via private communication) that describe the simulated CH_4 velocity distributions. The incidence energy E_i and the FWHM parameter were determined through time-of-flight measurements, whereas the stream velocity v_0 and width parameter α were determined through a fit to the aforementioned parameters using Eq. S4.

T_n (K)	$\langle E_\mathrm{i} \rangle$ (kJ/mol)	FWHM (kJ/mol)	v_0 (m/s)	α (m/s)
323	9.7	2.1	1093	72
323	22.0	3.0	1652	68
323	32.5	3.2	2011	60
323	44.5	5.0	2352	80
323	53.2	6.2	2571	90
373	63.4	8.4	2806	112

Table S4: Molecular beam parameters from Ref.[1] that describe the simulated CHD_3 velocity distributions. The stream velocity v_0 and width parameter α were determined through time-of-flight measurements

T_n (K)	$\langle E_\mathrm{i} \rangle$ (kJ/mol)	v_0 (m/s)	α (m/s)
400	81.7	2899	216
450	89.2	3026	246
500	97.4	3157	270
550	102.5	3231	299
600	111.9	3369	333
650	120.0	3483	367
