# Supplementary material for: Accurate Reaction Probabilities for Translational Energies on Both Sides of the Barrier of Dissociative Chemisorption on Metal Surfaces
Source: J Phys Chem Lett. 2024 Feb 28;15(9):2566–72. doi: 10.1021/acs.jpclett.3c03408 (PMC10926167; doi:10.1021/acs.jpclett.3c03408)
Supplement: Supplementary file 2 — jz3c03408_si_002.pdf [file jz3c03408_si_002.pdf]

jz-2023-03408y.R1

Name: Peer Review Information for "Accurate Reaction Probabilities for Translational Energies on Both Sides of the Barrier of Dissociative Chemisorption on Metal Surfaces"

First Round of Reviewer Comments

Reviewer: 1

Comments to the Author

The authors presented a new RPMD approach, which, for the first time, comprehensively incorporates the degrees of freedom associated with a mobile metal surface. This approach successfully replicates experimental sticking probabilities observed in the dissociative chemisorption of methane on Pt(111) across a large range of translational and vibrational energies. The simulations, characterized by the inclusion of nuclear quantum effects, contribute to a more accurate representation of gas-surface reactions. I would recommend its publication after the authors address the following comments.

1. In some places, abbreviations such as NVT and NVE are used. However, these terms should be spelled out in full the first time they appear.

2. It seems at even lower translational energy (<30 kJ/mol), the RPMD results can be substantially larger than the experimental results. I suggest the authors include those RPMD results for the translational energy below 30 kJ/mol and plot them together. Some discussions are also useful.

Reviewer: 2

Comments to the Author

In this work, the authors compute dissociative sticking probabilities of methane on Pt(111) by using the RPMD approach for non-equilibrium conditions. Such an approach has been recently applied to approximately treat quantum effects in surface reaction simulations in the rigid surface limit. Here, the key improvement is that surface degrees of freedom are included in RPMD simulations by using

a high-dimensional neural network potential. Under the “laser off” condition, the RPMD results seem in better agreement with experimental data in the middle range of incidence energies than standard QCT results. These results are also compared with those obtained by a reaction path Hamiltonian based quantum dynamics approach.

This topic is of course urgent and significant. Unfortunately, I do not see conceptually different results in this work compared with previous studies for H2 and D2O dissociative chemisorption. In addition, the main text contains one Figure only, and the discussion on the comparison is too limited to provide sufficiently important insights. I cannot recommend the manuscript of the current form for the publication of JPC Letters, unless the authors can address the following concerns in the resubmission.

A. While including surface DOFs in RPMD simulations represents an advancement, it is technically straightforward due to the trajectory-based formalism of RPMD and the well-established feasibility of HDNNP. In Figure 1, a similar reduction in sticking probabilities by RPMD compared to QCT is observed, as previously found for D2O/Ni(111) on a rigid surface. This just validates the ability of RPMD to preserve zero-point energy. However, the authors honestly acknowledged the limitations of RPMD in calculating sticking probabilities under realistic molecular beam conditions and/or specific vibrational states, which narrows the scope of its useful applications in molecule-surface reactions and thus weakens the general interest of this work to the community.

B. An interesting question raised by Figure 1 is regarding the impact of quantum mechanical treatment, albeit approximate, of surface DOFs in RPMD on reactivity. However, there does not seem to be any discussion addressing this point in the manuscript. It would also be valuable to explore whether the surface motion influences the leakage of zero-point energy (ZPE). I suggest that the authors include a comparison of sticking probabilities on a frozen surface alongside their current results. This additional analysis would allow for a discussion on potential quantum effects resulting from lattice motion.

C. Following the point B, the comparison in Figure 1 should be taken with caution. RPH is used here as a benchmark of quantum dynamics. While it is an elegant approach that clearly elaborates the vibrational mode specificity in the dissociate polyatomic molecules on surfaces, it relies on the harmonic approximation of vibrational modes and is not a fully-coupled solution. Additionally, the sudden-averaging treatment of lattice effects in RPH differs significantly from the direct MD-based treatment in RPMD and QCT. Furthermore, it is unclear whether and how the RPH results account for velocity distribution at each incidence energy. On the other hand, experimental results cannot serve as a benchmark either for the assessment of the intrinsic accuracy of different theories. This is because none of the theoretical models perfectly replicates the exact experimental conditions and is free of approximations. A good agreement between theory and experiment may result from

error cancellations. Consequently, accurate full quantum benchmark results with a moving surface are still missing, which prevents a definitive assessment of the RPMD performance in this work.

D. More serious issues appear in the RPMD setup. An effective temperature of 1000 K is imposed in the ring polymer Hamiltonian for the entire incidence energy range. However, for incidence energies below the minimum barrier height, the maximum nozzle temperature is much lower than 1000 K (say 323 K). This ad-hoc choice of temperature is not justified. It is hard to imagine that 323 K and 1000 K will lead to similar results at low energies. More confusingly, it is claimed that the surface temperature is fixed at 500 K in all simulations. It is not clear that what exactly the temperature of the system is during the collisional process (non-equilibrium condition). Furthermore, the initial rotational motion of the impinging molecule is removed in the RPMD setup, but in experiments the rotational temperature is typically on the order of dozens of Kelvin. This mismatch may also have an impact on the comparison of theoretical and experimental sticking probabilities. The authors provide some brief explanations but do not show any convincing numerical results in these aspects. This is not satisfying as a good performance of RPMD is the core of this work.

The material may become publishable as a JPCC paper provided points A and B are fixed. Should the authors also be able to address points C and D, then the manuscript might be publishable in a high impact journal like JPCL.

Minor points:

1. In the abstract and conclusion, the authors claim that "we introduce a ring polymer molecular dynamics approach that includes for the first time fully the degrees of freedom of a moving metal surface." But to me, this work is an application of the existing RPMD approach to model dissociative adsorption of a molecule on a moving surface. I suggest rephrase these statements. In addition, the recent paper of Li and Jiang on using the RPMD rate theory to compute the NO desorption rate constants should be referenced (Chem. Sci. 14, 5087, 2023).

2. QCT and RPMD results for lower energies should be present. Following the current trend, it seems that RPMD would unavoidably overestimate the sticking probability. Discussion on this range is necessary.

Author's Response to Peer Review Comments:

Reviewer comments are in black, our comments in blue, text from the original manuscript in purple, and changes to the manuscript in red.

Reviewer: 1

Recommendation: This paper may be publishable, but major revision is needed; I would like to be invited to review any future revision.

Comments:
The authors presented a new RPMD approach, which, for the first time, comprehensively incorporates the degrees of freedom associated with a mobile metal surface. This approach successfully replicates experimental sticking probabilities observed in the dissociative chemisorption of methane on Pt(111) across a large range of translational and vibrational energies. The simulations, characterized by the inclusion of nuclear quantum effects, contribute to a more accurate representation of gas-surface reactions. I would recommend its publication after the authors address the following comments.

We thank the reviewer for their positive evaluation and comments.

1A. In some places, abbreviations such as NVT and NVE are used. However, these terms should be spelled out in full the first time they appear.

NVT and NVE are not abbreviations, per se, but standard terms in thermodynamics. The letters do not always exactly relate to a word and therefore we cannot spell them out. We have checked and, to the best of our knowledge, all abbreviations other than NVT and NVE are spelled out the first time they appear. Instead, we have opted to indicate even more clearly what those terms mean as follows:

P6: "The vibrational initial conditions of the molecule are obtained by performing canonical NVT (constant number of particles, volume, and temperature) simulations in the gas phase ... Finally, we perform microcanonical NVE (constant number of particles, volume, and energy) simulations to simulate DC"

1B. It seems at even lower translational energy (<30 kJ/mol), the RPMD results can be substantially larger than the experimental results. I suggest the authors include those RPMD results for the translational energy below 30 kJ/mol and plot them together. Some discussions are also useful.

Here we answer both 1B and 2F. The experimental sticking probability S0 at the lowest incidence energy for which we performed RPMD calculations is 10^{-4}. The experimental S0 at the next nearest lower incidence energy is $4x10^{-6}$. To obtain any trajectories that involve sticking, one would need to run at least 1 million trajectories. If one also wants to have reasonable statistics (i.e., error bars that are comparable to experiment), one would even need to run 10-100 million trajectories. For QCT this is already a considerable computational investment (and would not yield a meaningful result). However, for RPMD we have used a smaller time step (0.1 vs 0.4 fs) and have to evaluate the forces for each individual bead (total of 28 beads), making the RPMD calculations in this case two orders of magnitude more expensive than QCT. For these reasons, it is intractable (for now) to perform RPMD calculations for the requested incidence energies. Nevertheless, we can still discuss how RPMD is expected to perform in that range, as we now do as follows:

P8: "Although, the RPMD calculations are considerably cheaper than QD, in this work they are still two orders of magnitude more expensive than QCT. Since calculations for lower incidence energies than performed here would also require considerably more trajectories (10^6-10^8 instead of 10^4), it is intractable at present to compute RPMD sticking probabilities for lower incidence energies than those presented here.

Future developments in machine-learned potentials and RPMD techniques combined with a general increase in computational resources should enable investigation of RPMD sticking probabilities at even lower incidence energies. Nevertheless, we can discuss how RPMD is expected to perform at incidence energies far below the minimum barrier height. For QCT, it is clear that the reactivity for translational energies below the minimum barrier height is always vastly overestimated due to artificial leakage of the ZPE into the reaction coordinate. So far, RPMD is shown to preserve the ZPE during the reaction much better than QCT, making accurate predictions of the sticking probability at low incidence energies possible. At E_i=33 kJ/mol, RPMD overestimates the experimental sticking slightly, but it should also be noted that the experimental result still falls well within the 1σ confidence interval of the RPMD result (the RPMD statistics here are severely limited, with only 4 trajectories out of 20000 having reacted). Previous results also indicate that in general RPMD yields accurate reaction rates even in the deep tunneling regime.[78-82] Moreover, the reactivity of methane at lower incidence energies is dominated by trajectories in which the molecule encounters a surface configuration that lowers the local barrier height considerably, and not by tunneling or energy exchange between the molecule and metal surface.[7,11,60,67,68,83] In fact, RPMD calculations employing a static ideal surface (i.e., the so-called Born-Oppenheimer static surface (BOSS) approximation, which approximates a T_s=0 K surface but still includes the thermal lattice expansion corresponding to T_s=500 K) yielded at E_i=33 kJ/mol no reactive trajectories out a total of 20000, in good agreement with previous experimental and theoretical results showing considerable increase in sticking at low incidence energy with the surface temperature.[61,63,65,83–85] Exploratory calculations also suggest that the BOSS results match the moving surface results at higher incidence energies above the minimum barrier height, again in agreement with experiment and theory. In short, both ZPE conservation and surface atom motion need to be described correctly in order to provide accurate simulations for CH_4 + Pt(111) at incidence energies below the minimum barrier height, which seems to be the case here. For these reasons, we expect RPMD to reproduce the experiments at even lower incidence energies as well. We hope that the aforementioned developments will allow for testing this hypothesis in the future."

Additional Questions:
Urgency: High

Significance: High

Novelty: High

Scholarly Presentation: High

Is the paper likely to interest a substantial number of physical chemists, not just specialists working in the authors' area of research?: Yes

Reviewer: 2

Recommendation: This paper may be publishable, but major revision is needed; I would like to be invited to review any future revision.

Comments:
In this work, the authors compute dissociative sticking probabilities of methane on Pt(111) by using the RPMD approach for non-equilibrium conditions. Such an approach has been recently applied to approximately treat quantum effects in surface reaction simulations in the rigid surface limit. Here, the key improvement is that surface degrees of freedom are included in RPMD simulations by using a highdimensional neural network potential. Under the "laser off" condition, the RPMD results seem in better agreement with experimental data in the middle range of incidence energies than standard QCT results. These results are also compared with those obtained by a reaction path Hamiltonian based quantum dynamics approach.

This topic is of course urgent and significant. Unfortunately, I do not see conceptually different results in this work compared with previous studies for H2 and D2O dissociative chemisorption. In addition, the main text contains one Figure only, and the discussion on the comparison is too limited to provide sufficiently important insights. I cannot recommend the manuscript of the current form for the publication of JPC Letters, unless the authors can address the following concerns in the resubmission.

We thank the reviewer for their critical evaluation and comments. However, in our opinion, the inclusion of surface DOFs makes this work conceptually significantly different from the previous work on H2 and D2O. If we would have excluded surface atom motion, our results would not have reproduced the experimental or
RPH results at low incidence energy at all. This work constitutes the first time that anyone has performed an RPMD study on a moving metal surface, and is therefore an important test case for the validity of the method. We have extended the discussion in the new manuscript considerably. Although there is only a single figure, it does consist out of two subpanels since plotting the data together gives a better overview. We have looked at similar letters in JPCL, and the amount of content in our figure ends up not being different from many of them. Even more so since we have moved figures that are not so relevant for the main discussion to the SI, in order to increase the clarity and focus on the significant and novel aspects of our work. We believe to have addressed all the reviewer's concerns, making the publication suitable for JPCL.

2A. While including surface DOFs in RPMD simulations represents an advancement, it is technically straightforward due to the trajectory-based formalism of RPMD and the well-established feasibility of HDNNP. In Figure 1, a similar reduction in sticking probabilities by RPMD compared to QCT is observed, as previously found for D2O/Ni(111) on a rigid surface. This just validates the ability of RPMD to preserve zero-point energy.

As already mentioned in the original manuscript, accurate simulations of the dissociative chemisorption of methane require also surface atom motion, not just ZPE conservation. Our responses to 1B and 2B show that we now emphasize more clearly in the new manuscript that both ZPE conservation and surface atom motion are necessary and described well, as well as discussing the effect of surface atom motion on the sticking probability more thoroughly.

However, the authors honestly acknowledged the limitations of RPMD in calculating sticking probabilities under realistic molecular beam conditions and/or specific vibrational states, which narrows the scope of its useful applications in molecule-surface reactions and thus weakens the general interest of this work to the community.

We are afraid we do not understand this remark of the reviewer. We indeed acknowledged that at present there is no reliable method to produce vibrational state-specific RPMD sticking results. However, the results presented are for several different molecular beams containing different Boltzmann vibrational state distributions, which illustrates the opposite: RPMD can compute sticking probabilities for realistic molecular beam conditions involving Boltzmann vibrational distributions, which are also highly relevant for catalytic conditions and pertain to the vast majority of catalytically relevant simulations. We simply provide the outlook that the development of a method for vibrational state-specific initial conditions would extend the RPMD method to the full capabilities of QCT. But even in the absence of such a method, RPMD simulations can provide in the vast majority of catalytically relevant simulations meaningful results, where we again emphasize that QCT cannot do this. In other words, this work should be relevant for the entire community interested in dynamical simulations related to (heterogeneous) catalysis. To reflect this, we made the following change:

P10: "This is excellent when comparing to supersonic molecular beam experiments under "laser-off" conditions or other catalytically relevant experiments, since the vibrational state distribution is the same as the one we simulate."

2B. An interesting question raised by Figure 1 is regarding the impact of quantum mechanical treatment, albeit approximate, of surface DOFs in RPMD on reactivity. However, there does not seem to be any discussion addressing this point in the manuscript. It would also be valuable to explore whether the surface motion influences the leakage of zero-point energy (ZPE). I suggest that the authors include a comparison of sticking probabilities on a frozen surface alongside their current results. This additional analysis would allow for a discussion on potential quantum effects resulting from lattice motion.

We realize that we did not indicate clearly enough how much the surface atom motion affects our results. We have now added a more elaborate discussion. However, as we also mentioned in our response to 1B, the RPMD calculations are quite expensive, especially when dealing with very low sticking probabilities. We have performed exploratory calculations investigating the effect of keeping the surface atoms fixed in their ideal positions, i.e., a static ideal surface (commonly referred to as the "Born-Oppenheimer static surface" (BOSS) approximation). We have opted to not include the numerical results in the paper since they are statistically much more limited and we do not believe that much can be learned from these results. For E_i=33 kJ/mol we saw zero reacted trajectories (i.e., BOSS underestimates sticking compared to a moving surface), whereas for larger incidence energies the RPMD BOSS results match the moving surface results. This is a well-known phenomenom for the DC of methane on metal surfaces. Surface atom motion does not affect the reactivity much for incidence energies above the minimum barrier height. For incidence energies below the minimum barrier height, surface atom motion simply cannot be neglected since its effect dominates the reactivity in comparison to T_s=0 K. We have now added additional discussion regarding the surface DOFs as follows:

P3: "Also, due to surface atom motion, energy transfer between the molecule and the metal surface as well as temperature-dependent barrier height modulation can affect the reactivity considerably.[7,10,11]"

P4: "Unfortunately, such calculations are even more expensive than static surface calculations and it is unclear how accurate they are in describing a fully moving surface interacting with a (polyatomic) molecule, where surface atom motion is often a non-negligible effect."

P5: "Specifically, we choose the DC of methane on Pt(111) as a test case for our approach as the reaction dynamics are well understood[59,60], a large amount of experimental data is available to benchmark theory[27,61–66], and it is a system where the ZPE and surface atom motion play an important and non-negligible role in the reactivity.[7,43,60,67,68]"

P8: "Moreover, the reactivity of methane at lower incidence energies is dominated by trajectories in which the molecule encounters a surface configuration that lowers the local barrier height considerably, and not by tunneling or energy exchange between the molecule and metal surface.[7,11,60,67,68,83] In fact, RPMD calculations employing a static ideal surface (i.e., the so-called Born-Oppenheimer static surface (BOSS) approximation, which approximates a T_s=0 K surface but still includes the thermal lattice expansion corresponding to T_s=500 K) yielded at E_i=33 kJ/mol no reactive trajectories out a total of 20000, in good agreement with previous experimental and theoretical results showing considerable increase in sticking at low incidence energy with the surface temperature.[61,63,65,83–85] Exploratory calculations also suggest that the BOSS results match the moving surface results at higher incidence energies above the minimum barrier height, again in agreement with experiment and theory. In short, both ZPE conservation and surface atom motion need to be described correctly in order to provide accurate simulations for CH_4 + Pt(111) at incidence energies below the minimum barrier height, which seems to be the case here."

2C. Following the point B, the comparison in Figure 1 should be taken with caution. RPH is used here as a benchmark of quantum dynamics. While it is an elegant approach that clearly elaborates the vibrational mode specificity in the dissociate polyatomic molecules on surfaces, it relies on the harmonic approximation of vibrational modes and is not a fully-coupled solution. Additionally, the sudden-averaging treatment of lattice effects in RPH differs significantly from the direct MD-based treatment in RPMD and QCT. Furthermore, it is unclear whether and how the RPH results account for velocity distribution at each incidence energy. On the other hand, experimental results cannot serve as a benchmark either for the assessment of the intrinsic accuracy of different theories. This is because none of the theoretical models perfectly replicates the exact experimental conditions and is free of approximations. A good agreement between theory and experiment may result from error cancellations. Consequently, accurate full quantum benchmark results with a moving surface are still missing, which prevents a definitive assessment of the RPMD performance in this work.

The point of this work is not to use RPMD to treat a model system that is sufficiently simple to allow for a comparison with an exact quantum calculation. As the reviewer notes, that has been done for H2O dissociation on a static surface, but it should also be noted that this was only possible for a single rovibrational state with a limited computational setup. Our desire is to determine whether RPMD can provide an accurate MD-based description of dissociative chemisorption in a real system, i.e., whether it can be compared directly with experiment. CH4 is an obvious choice for a molecule, given the industrial/societal relevance and the large amount of experimental data available, but an exact quantum solution is not remotely possible, even for dissociative chemisorption on a static surface. Moreover, QCT-based studies at high energy and RPH-based studies at low energy clearly show that the addition of lattice motion significantly modifies the reaction probability, and its inclusion is essential for any elucidation of the experimental data (see also, e.g., our response to 2B).

Simple exactly soluble models are useful, but approximate models of complex systems are also useful. So, what does one do? Clearly, the only options are to compare with experiment and any other approximate theories that have been shown to be reasonably accurate. While the RPH does include a harmonic approximation, this is used only for the more strongly bound (higher frequency) modes, and yes, the lattice effects are introduced in an approximate and different way from the RPMD treatment. Part of our motivation for this study is that RPMD avoids both of these approximations. Having said this, the RPH approach, combined with the SRP density functional, has led to surprisingly good agreement with molecular beam studies of CH4 on several Ni, Pt and Ir surfaces. Unfortunately, a rigorous quantum treatment of this important reaction will likely not occur in our lifetimes, but experiments and approximate models, and the agreement between them, will continue to improve. In addition to the original manuscript and the various changes outlined in the rest of our rebuttal, we emphasize this point now even more as follows:

P4: "Accurate wave packet quantum dynamics (QD) do include NQEs, but generally scale badly with the number of degrees of freedom (DOFs) on top of the already considerably higher computational cost compared to QCT, severely limiting the number of DOFs and the quality of the QD basis set that can be treated, also in the foreseeable future.[38-40]"

P9: "We have also included reaction path Hamiltonian[83,87,88] (RPH) results in Figure 1 as a QD benchmark for RPMD, given the method's success in simulating reactivity of methane on several metal surfaces.[10,27,89,90]"

Regarding the Reviewer's comment about the molecular beam velocity distribution, the wave packet based RPH method gives dissociation probabilities that are resolved with respect to both vibrational state and incidence energy (or velocity). Given this, it is easy to average over the incidence velocity distribution and vibrational state distributions described in the original SI (see also the RPH references provided in the main manuscript for the computational details). We also note that we already referred the reader to Migliorini et al. for details regarding the RPH calculations since they have already been fully described.

2D. More serious issues appear in the RPMD setup. An effective temperature of 1000 K is imposed in the ring polymer Hamiltonian for the entire incidence energy range. However, for incidence energies below the minimum barrier height, the maximum nozzle temperature is much lower than 1000 K (say 323 K). This adhoc choice of temperature is not justified. It is hard to imagine that 323 K and 1000 K will lead to similar results at low energies.

Sure, a temperature of 323 K would be definitely be too low for the entire system. Both the surface atom motion and translational motion of the molecule are larger than 323 K (the former is 500 K, the latter is illdefined due to it not being a Maxwell-Boltzmann distribution but a different flux-weighted distribution; see SI), so it also wouldn't make sense to use such a low effective temperature. Like we already mentioned in the original discussion, there simply is not a single temperature. Therefore, one has to make a choice. Again, it is not clear, yet, how to make this choice. Nevertheless, the work of Marjolet and co. suggests that the amount of translational energy should be very important in determining the temperature. As such, it is reasonable to expect that the effective temperature should be at least 500 K (due to the surface atom motion/temperature together with the high translational energies compared to a Maxwell-Boltzmann distribution at a similar temperature) and tests have indicated that further increase of the temperature doesn't matter (much). This is also expected from the RPMD Hamiltonian in combination

with the sticking probability dependence of methane with various NQEs and temperature: At low incidence energy sticking is dominated by surface atom motion effects, not tunneling. QCT can already describe the surface atom motion reasonably well, as evidenced by previous studies, but it cannot contain the ZPE correctly in the right coordinates. As we have already indicated numerous times, RPMD solves especially the ZPE violation problem. Since we are actually not so concerned with tunneling, the effective temperature should also have less of an effect on the sticking probability, which is corroborated by our exploratory calculations regarding the effective temperature (P11:
"Fortunately, in our case the computed results do not seem to be very dependent on the choice of temperature, as long as the effective temperature is higher than that of the surface (T_s = 500 K).").

More confusingly, it is claimed that the surface temperature is fixed at 500 K in all simulations. It is not clear that what exactly the temperature of the system is during the collisional process (non-equilibrium condition).

We did not "fix" the surface temperature in all simulations, since we have performed both NVT and NVE simulations. Obviously, in the NVT simulations the surface temperature is fixed, but in the NVE simulation it is not. What we did do, is only simulate Ts=500 K. This means that the initial surface atom positions and momenta correspond to Ts=500 K. But during the reactive scattering simulations (i.e., NVE), the only constraint present is that the total energy of the system is constant. This also means that the surface temperature is no longer fixed. As long as the molecule and surface do not interact, the temperature of the surface is stable. But as soon as the two interact, energy can be transferred to or from the surface DOFs and therefore the temperature can change, i.e., no constraint on the surface temperature is placed and thus it is not "fixed". The reviewer is right in saying that the temperature during the collisional process is unknown, which is always the case, regardless of employed computational approach. But this is not a problem for the nonequilibrium NVE simulations (see also above). These facts were already discussed extensively in the original manuscript (see P6-7 and Section S3) and improved further in clarity (see our response to 1A).

Furthermore, the initial rotational motion of the impinging molecule is removed in the RPMD setup, but in experiments the rotational temperature is typically on the order of dozens of Kelvin. This mismatch may also have an impact on the comparison of theoretical and experimental sticking probabilities. The authors provide some brief explanations but do not show any convincing numerical results in these aspects. This is not satisfying as a good performance of RPMD is the core of this work.

We believe that the reviewer misunderstood the procedure (also note that the procedure regarding the vibrational motion is the same as in Ref. 56). Rotational motion is only removed from the simulations from which we extract the vibrational initial conditions, both translational and rotational motion are added in a later stage but before initiating the reactive scattering simulations. However, for the reactive scattering simulations we still include the rotational motion as is appropriate for the simulated molecular beam. Or in this particular case, we only include the rotational orientation since we simulate J=0. Furthermore, it is an accepted fact that the rotational state does not affect sticking probabilities of methane significantly (see, e.g., Juurlink et al, DOI: 10.1039/B003708G), and therefore does not require additional numerical evidence from us. This was already described in the manuscript and SI, and now further clarified, as follows:

P6: For the RPMD, we take a similar approach as Ref. [57], but here the surface atom motion is included as well. The vibrational initial conditions of the molecule are obtained by performing canonical NVT

(constant number of particles, volume, and temperature) simulations in the gas phase with the so-called PIGLET approach,[74,75] of which any translational and rotational motion is removed afterwards since from these simulations we only require the vibrational positions and moments. … Finally, we perform microcanonical NVE (constant number of particles, volume, and energy) simulations to simulate DC, by first adding translational and rotational motion to the molecule's center of mass as well as reorienting the molecule according to its rotational state (see for example Chapter 2 of Ref. [76]).

PS7: Moreover, the simulated rotational state of CH_4 and CHD_3 is J=0 (i.e., the rotational ground state), which is appropriate for the simulation of supersonic molecular beams of methane[1,32].

The material may become publishable as a JPCC paper provided points A and B are fixed. Should the authors also be able to address points C and D, then the manuscript might be publishable in a high impact journal like JPCL.

We believe that we have addressed all concerns of the reviewer. Furthermore, we again point out that inclusion of NQEs in high-dimensional simulations of dissociative chemisorption on metal surfaces is not trivial. The importance of a dynamical (approximate) approach that is able to include both NQEs and surface atom motion and that reproduces other established theoretical and experimental benchmarks on moleculemetal surface cannot be understated.

Minor points:
2E. In the abstract and conclusion, the authors claim that “we introduce a ring polymer molecular dynamics approach that includes for the first time fully the degrees of freedom of a moving metal surface.” But to me, this work is an application of the existing RPMD approach to model dissociative adsorption of a molecule on a moving surface. I suggest rephrase these statements. In addition, the recent paper of Li and Jiang on using the RPMD rate theory to compute the NO desorption rate constants should be referenced (Chem. Sci. 14, 5087, 2023).

We have rephrased the sentence (“Here, we extend a ring polymer molecular dynamics approach to include for the first time fully the degrees of freedom of a moving metal surface.”) and included the reference.

2F. QCT and RPMD results for lower energies should be present. Following the current trend, it seems that RPMD would unavoidably overestimate the sticking probability. Discussion on this range is necessary.

We actually do not expect that RPMD would overestimate the sticking probability considerably. We have replied to this comment (2F) in full in our response to 1B.

Additional Questions:
Urgency: High

Significance: High

Novelty: Moderate

Scholarly Presentation: Moderate

Is the paper likely to interest a substantial number of physical chemists, not just specialists working in the authors' area of research?: No

jz-2023-03408y.R2

Name: Peer Review Information for "Accurate Reaction Probabilities for Translational Energies on Both Sides of the Barrier of Dissociative Chemisorption on Metal Surfaces"

Second Round of Reviewer Comments

Reviewer: 1

Comments to the Author

I suggest the acceptance of this manuscript.

Reviewer: 2

Comments to the Author

The authors have partially addressed my concerns and included more discussions in the revision, which I appreciate very much. However, they do not present any additional results as both reviewers requested. Let me emphasize some unsolved points as follows:

1. The authors argue that the experimental sticking probability at 22 kJ/mol is 4x10^-6 so that RPMD calculations were too expensive to reach such a low reactivity. However, the problem is that the RPMD result at 33 kJ/mol starts to decrease less significantly than experimental data. This is why both reviewers had the impression that RPMD results at lower energies may overestimate the reactivity and wanted to see some more low energy data to understand this trend. It is unnecessary to perform QCT and RPMD calculations down to 22 kJ/mol, but one or two additional data points, say at 30 kJ/mol and/or 27kJ/mol, will be useful to check if the RPMD result followed the trend or not.

2. The authors argue that their desire is to determine whether RPMD can provide an accurate description of dissociative chemisorption that can be compared directly with experiment under typical molecular beam conditions, rather than to benchmark this approach with exact quantum calculation. This is indeed an important goal. But to make RPMD a general approach for this goal, the choice of effective temperature and initial sampling has to be justified to make sure that the

agreement with experiment is not a coincidence. In this respect, I do not feel that my concerns are adequately addressed.

There are several different temperatures in a realistic simulation of dissociative chemisorption, representing intrinsic difficulties in the RPMD calculation. In QCT, one can easily sample the vibrational and angular momenta of the molecule according to vibrational and rotational temperatures, respectively. The translational energy distribution does not correspond to a specific temperature but follows the flux distribution given in the SI. The surface configurations are sampled in an equilibrated NVT simulation for a given surface temperature. Once initial conditions are determined, the trajectory is propagated in NVE without the need for defining a temperature.

However, in RPMD, there is an effective temperature associated with the frequency of the beads, which appears in both sampling (NVT) and propagation (NVE). It is now clear that the surface is initially sampled at 500 K, so I suppose this temperature is assigned to these beads representing surface atoms. However, many details remain ambiguous. a) what the vibrational temperature is applied for the sampling of vibrational momenta for the molecule? Does it correspond to the nozzle temperature at each incidence energy? This temperature varies with the incidence energy. b) the rotational temperature seems zero (J=0 or rotational angular momenta are removed during sampling), but the original SI mentions that the J=2 state of CHD_3 is simulated. c) Most importantly, when propagating the non-equilibrium RPMD trajectory, the effective temperature for the entire molecule-surface system seems to be elevated to 1000 K, if I understood it correctly. At least two issues exist here. First, how does this sudden change of effective temperature affect the dynamics? In this case, the sampled equilibrium condition will be destroyed immediately. Second, why 1000 K? this temperature does not correspond to any physical temperature of this system. The authors should provide numerical results showing the dependence on the choice of temperature. To me, increasing the effective temperature from 500 K to 1000 K will make a big difference in the total energy. It is surprising that they will not change the calculated sticking probability, especially at low energies where the absolute value is low. In addition, as the authors mentioned, the amount of translational energy should be very important in determining the temperature. It is very likely the simulations at different incidence energies should rely on different effective temperatures, as implemented in the work of Marjolet and coworkers. This also requires the presence of temperature-dependent results and more discussions in the manuscript.

To conclude, I emphasize that these technical details are critical in supporting the significance of this work. After all, the effective temperature is a unique problem for the non-equilibrium RPMD simulation that is not present in QCT and quantum calculations. If only a specific protocol of RPMD can reproduce experiments and the choice of effective temperature is system-dependent, how should one use the RPMD approach to describe dissociation chemisorption in other systems?

Author's Response to Peer Review Comments:

Reviewer comments are in black, our comments in blue, text from the original manuscript in purple, and changes to the manuscript in red.

Reviewer(s)' Comments to Author:

Reviewer: 1

Recommendation: This paper represents a significant new contribution and should be published as is.

Comments:
I suggest the acceptance of this manuscript.

Additional Questions:
Urgency: High

Significance: High

Novelty: High

Scholarly Presentation: High

Is the paper likely to interest a substantial number of physical chemists, not just specialists working in the authors' area of research?: Yes

Reviewer: 2

Recommendation: This paper may be publishable, but major revision is needed; I would like to be invited to review any future revision.

Comments:
The authors have partially addressed my concerns and included more discussions in the revision, which I appreciate very much. However, they do not present any additional results as both reviewers requested. Let me emphasize some unsolved points as follows:

1. The authors argue that the experimental sticking probability at 22 kJ/mol is 4x10^-6 so that RPMD calculations were too expensive to reach such a low reactivity. However, the problem is that the RPMD result at 33 kJ/mol starts to decrease less significantly than experimental data. This is why both reviewers had the impression that RPMD results at lower energies may overestimate the reactivity and wanted to see some more low energy data to understand this trend. It is unnecessary to perform QCT and RPMD calculations down to 22 kJ/mol, but one or two additional data points, say at 30 kJ/mol and/or 27kJ/mol, will be useful to check if the RPMD result followed the trend or not.

We appreciate the reviewer's desire to evaluate the quality of our RPMD approach at even lower incidence energies. However, in order to perform the calculations suggested by the reviewer, one would need to make a guess for the molecular beam parameters, since experimental parameters would not be available. This would introduce some uncertainty in the comparison with experiment, in addition to the experimental sticking probability being unknown, thus lacking a reliable benchmark. Furthermore, since the sticking probability will drop with a decreasing incidence energy, the statistics will become worse, even with the smaller step in incidence energy. Thus, instead of the previously mentioned 10-100 million RPMD trajectories at Ei=22

kJ/mol, one would likely still need ~100k-1 million trajectories. For our RPMD simulations, this is equivalent to running 11-114 million QCTs on a high-dimensional PES, which would be extremely costly and intractable. Note that the only existing study of methane reacting on a high-dimensional machine-learned potential so far, which is also still a fairly recent paper (10.1021/acs.jpclett.9b00560), performed 10000-110000 QCTs at any given incidence energy. The requested single data point would require a similar computational cost as was required for all our current data. Furthermore, we believe that the reviewer is incorrect in stating that the RPMD starts to decrease less significantly. In fact, comparing Ei=33 and 45 kJ/mol might suggest the opposite: At Ei=33 kJ/mol the experimental result falls within the 68% confidence interval of RPMD (P8: "At Ei=33 kJ/mol, RPMD overestimates the experimental sticking slightly, but it should also be noted that the experimental result still falls well within the 1σ confidence interval of the RPMD result..."), whereas at Ei=45 kJ/mol the experimental result falls just outside of this interval. Additionally, we have extensively discussed how the approach is expected to behave at even lower incidence energies at P8-9. We emphasize that reviewer 1 is convinced by our previous arguments regarding this point, and required no additional work from us, recommending to publish our work as is. For these reasons, we believe that it is not possible, nor that there is need, to perform new RPMD calculations at lower incidence energies.

2. The authors argue that their desire is to determine whether RPMD can provide an accurate description of dissociative chemisorption that can be compared directly with experiment under typical molecular beam conditions, rather than to benchmark this approach with exact quantum calculation. This is indeed an important goal. But to make RPMD a general approach for this goal, the choice of effective temperature and initial sampling has to be justified to make sure that the agreement with experiment is not a coincidence. In this respect, I do not feel that my concerns are adequately addressed.

There are several different temperatures in a realistic simulation of dissociative chemisorption, representing intrinsic difficulties in the RPMD calculation. In QCT, one can easily sample the vibrational and angular momenta of the molecule according to vibrational and rotational temperatures, respectively. The translational energy distribution does not correspond to a specific temperature but follows the flux distribution given in the SI. The surface configurations are sampled in an equilibrated NVT simulation for a given surface temperature. Once initial conditions are determined, the trajectory is propagated in NVE without the need for defining a temperature.

However, in RPMD, there is an effective temperature associated with the frequency of the beads, which appears in both sampling (NVT) and propagation (NVE). It is now clear that the surface is initially sampled at 500 K, so I suppose this temperature is assigned to these beads representing surface atoms.

We thank the reviewer for pointing out the remaining ambiguous details. We have addressed them as follows.

In our NVT RPMD simulations, the temperature in the Hamiltonian is equal to that of the thermostat, which is standard practice. We further clarify this as follows:

PS6: "The NVT simulations for the surface and molecular vibrational initial conditions employ the PIGLET approach[21,22], where the temperature in the RPMD Hamiltonian is always equal to that of the thermostat."

In our NVE RPMD simulations, the temperature in the Hamiltonian is taken to be 1000 K, which was already mentioned in the SI:

PS6: "In the NVE simulations, the system is no longer in equilibrium and therefore we need to make a choice for the effective temperature in the ring polymer Hamiltonian, here taken to be 1000 K."

However, many details remain ambiguous. a) what the vibrational temperature is applied for the sampling of vibrational momenta for the molecule? Does it correspond to the nozzle temperature at each incidence energy? This temperature varies with the incidence energy.

Indeed, the vibrational temperature and nozzle temperature are identical. The nozzle temperature at each incidence energy was already provided in the Tables S3 and S4, and the computational details (e.g., how to determine the vibrational temperature) are the same as in 10.1021/acs.jpclett.7b01905, which was already mentioned in the original manuscript ("The QCT simulations are performed in the same way as in Ref. 1 … As such, we will only discuss the details relevant to the RPMD simulations."). This is now further clarified as follows:

PS6: "… to obtain the vibrational positions and velocities. The vibrational temperature is equal to the nozzle temperature. These simulations are first equilibrated … "

b) the rotational temperature seems zero (J=0 or rotational angular momenta are removed during sampling), but the original SI mentions that the J=2 state of CHD3 is simulated.

We apologize that we forgot to mention this fact in our previous reply letter. The original SI erroneously stated that for CHD_3 J=2 is simulated in the NVE simulations. (We have always clearly stated that in the vibrational NVT simulations rotational motion is removed afterwards.) However, this is only true for laser-on conditions, which are not simulated in this work. Therefore, we only simulate J=0, which is appropriate for a methane molecular beam under laser-off conditions (see also the SI).

On a similar note, we have also discovered that the SI previously mentioned the use of 28 beads, but we used 24 beads instead (note that this does not affect any of the results or the discussion thereof). We have compared our input files and the computational details in the manuscript to confirm that no additional errors are present.

c) Most importantly, when propagating the non-equilibrium RPMD trajectory, the effective temperature for the entire molecule-surface system seems to be elevated to 1000 K, if I understood it correctly. At least two issues exist here. First, how does this sudden change of effective temperature affect the dynamics? In this case, the sampled equilibrium condition will be destroyed immediately.

Unfortunately, the reviewer's question is phrased rather broadly. For example, the intricacies of the ring dynamics themselves go far beyond the scope of this work. We have opted to extend the references regarding existing non-equilibrium RPMD work that involve more fundamental model systems (P7: "Fortunately, RPMD seems so far to be well-suited for not just equilibrium but for non-equilibrium simulations as well.[49,56,57,77–79]"). In general, these show that non-equilibrium RPMD performs well compared to traditional equilibrium RPMD.

Furthermore, a thorough analysis of the reaction dynamics of the molecule in the RPMD simulations would be uninteresting as they are the same as in QCT and RPH, within a certain extent, and are well understood for methane (which was already discussed in the manuscript).

Second, why 1000 K? this temperature does not correspond to any physical temperature of this system. The authors should provide numerical results showing the dependence on the choice of temperature. To me, increasing the effective temperature from 500 K to 1000 K will make a big difference in the total energy. It is surprising that they will not change the calculated sticking probability, especially at low energies where the absolute value is low. In addition, as the authors mentioned, the amount of translational energy should be very important in determining the temperature. It is very likely the simulations at different incidence energies should rely on different effective temperatures, as implemented in the work of Marjolet and coworkers. This also requires the presence of temperature-dependent results and more discussions in the manuscript.

The choice for 1000 K is an arbitrary one, although Marjolet et al. have shown for methane that this is likely to be a reasonable choice (note that here beta=312 a.u., whereas Marjolet et al. obtained, with their model, a range of 300-550 a.u., depending on the incidence energy and isotope). It is clear that in this work the Hamiltonian temperature should be larger than the vibrational temperature, since the temperatures associated with the surface atom motion and especially the molecular translation are considerably larger than the vibrational temperature. Unfortunately, it is not clear how one should partition the energy and obtain an effective temperature (this is also shown in multiple papers authored by Marjolet and co.). For a more realistic picture, one might even need to move away from a single temperature model and introduce a separate temperature for different partitions in the degrees of freedom. However, this goes far beyond the scope of our work. We already discussed these aspects in the manuscript, but they should be primarily viewed as important avenues of future work. Moreover, calculations by Li et al. (10.1080/00268976.2021.1941367) for H_2 + Pd(111) show that the choice of temperature (they compared T=300 K and 1052 K) hardly affected the sticking probability. This is a reaction that we expect to be considerably more affected by the effective temperature in the ring polymer Hamiltonian, due to the light mass of the hydrogen atoms, i.e., tunneling should play a much larger role than for methane. We now include this fact as follows:

P11: "...as long as the effective temperature is higher than that of the surface (Ts = 500 K). This is in agreement with Li et al., who observed hardly a difference for the sticking of H_2 on a static Pd(111) surface at T = 300 K and 1052 K.[100] Nevertheless, it
is clear that future work should..."

Furthermore, we have performed additional NVE RPMD calculations for low incidence energies that employ T=500 K in the ring Hamiltonian. These simulations reproduce well the results of the T=1000 K NVE RPMD simulations. The employed incidence energies are well below the minimum barrier height and therefore effects of the effective temperature should be well visible on the sticking probability. We believe that these numerical results support our earlier statements regarding the dependence of the sticking probability on the effective temperature. We have included these results in the new Figure S5 as follows:

P11: "Fortunately, in our case the computed results do not seem to be very dependent on the choice of temperature, as long as the effective temperature is higher than that of the surface (Ts=500 K; see Figure S5)."

PS6: "Figure S5 shows that, even for incidence energies well below the minimum barrier height, employing a lower effective temperature (500 K) hardly affects the sticking probability."

To conclude, I emphasize that these technical details are critical in supporting the significance of this work. After all, the effective temperature is a unique problem for the non-equilibrium RPMD simulation that is not present in QCT and quantum calculations. If only a specific protocol of RPMD can reproduce experiments and the choice of effective temperature is system-dependent, how should one use the RPMD approach to describe dissociation chemisorption in other systems?

Although there is some uncertainty of the sticking probability due to the effective temperature in the ring polymer Hamiltonian, this does not seem to matter too much quantitatively for the presented results. Qualitatively the same comparison is obtained: RPMD solves the ZPE violation problem present in QCT and reduces the artificial IVR problem, making RPMD more suitable than QCT for simulations performed at incidence energies both below and above the minimum barrier height. Therefore, we argue that the use of RPMD at any effective temperature yields more accurate results than QCT.

Additional Questions:
Urgency: High

Significance: High

Novelty: High

Scholarly Presentation: High

Is the paper likely to interest a substantial number of physical chemists, not just specialists working in the authors' area of research?: Yes
